# Functional Magnetic Resonance Imaging Evaluation of Auricular Percutaneous Electrical Neural Field Stimulation for Fibromyalgia: Protocol for a Feasibility Study

**DOI:** 10.2196/resprot.8692

**Published:** 2018-02-06

**Authors:** Melat Gebre, Anna Woodbury, Vitaly Napadow, Venkatagiri Krishnamurthy, Lisa C Krishnamurthy, Roman Sniecinski, Bruce Crosson

**Affiliations:** ^1^ Center for Visual and Neurocognitive Rehabilitation Research & Development Atlanta Veterans Affairs Medical Center Decatur, GA United States; ^2^ Division of Pain Medicine Department of Anesthesiology Emory University School of Medicine Atlanta, GA United States; ^3^ Martinos Imaging Center Massachusetts General Hospital Harvard University Charlestown, MA United States; ^4^ Department of Neurology Emory University Atlanta, GA United States; ^5^ Physics and Astronomy Georgia State University Atlanta, GA United States; ^6^ Division of Cardiothoracic Anesthesiology Department of Anesthesiology Emory University School of Medicine Atlanta, GA United States

**Keywords:** fibromyalgia, fMRI, PENFS, PENS, pain, sympathetic, auricular

## Abstract

**Background:**

Fibromyalgia is a chronic pain state that includes widespread musculoskeletal pain, fatigue, psychiatric symptoms, cognitive and sleep disturbances, and multiple somatic symptoms. Current therapies are often insufficient or come with significant risks, and while there is an increasing demand for non-pharmacologic and especially non-opioid pain management such as that offered through complementary and alternative medicine therapies, there is currently insufficient evidence to recommend these therapies. Percutaneous electrical neural stimulation (PENS) is an evidence-based treatment option for pain conditions that involves electrical current stimulation through needles inserted into the skin. Percutaneous electrical neural field stimulation (PENFS) of the auricle is similar to PENS, but instead of targeting a single neurovascular bundle, PENFS stimulates the entire ear, covering all auricular branches of the cranial nerves, including the vagus nerve. The neural mechanisms of PENFS for fibromyalgia symptom relief are unknown.

**Objective:**

We hypothesize that PENFS treatment will decrease functional brain connectivity between the default mode network (DMN) and right posterior insula in fibromyalgia patients. We expect that the decrease in functional connectivity between the DMN and insula will correlate with patient-reported analgesic improvements as indicated by the Defense and Veterans Pain Rating Scale (DVPRS) and will be anti-correlated with patient-reported analgesic medication consumption. Exploratory analyses will be performed for further hypothesis generation.

**Methods:**

A total of 20 adults from the Atlanta Veterans Affairs Medical Center diagnosed with fibromyalgia will be randomized into 2 groups: 10 subjects to a control (standard therapy) group and 10 subjects to a PENFS treatment group. The pragmatic, standard therapy group will include pharmacologic treatments such as anticonvulsants, non-steroidal anti-inflammatory drugs, topical agents and physical therapy individualized to patient comorbidities and preferences, prescribed by a pain management practitioner. The PENFS group will include the above therapies in addition to the PENFS treatments. The PENFS subject group will have the Neuro-Stim System placed on the ear for 5 days then removed and replaced once per week for 4 weeks. The primary outcome will be resting functional magnetic resonance imaging connectivity between DMN and insula, which will also be correlated with pain relief and functional improvements. This connectivity will be analyzed utilizing functional connectivity magnetic resonance imaging (fcMRI) and will be compared with patient-reported analgesic improvements as indicated by the DVPRS and patient-reported analgesic medication consumption. Pain and function will be further evaluated using Patient-Reported Outcomes Measurement Information System measures and measures describing a person’s functional status from Activity and Participation section of the International Classification of Functioning Disability and Health.

**Results:**

This trial has been funded by the Veterans Health Administration Program Office. This study attained approval by the Emory University/Veterans Affairs (VA) institutional review board and VA Research & Development committee. Institutional review board expedited approval was granted on 2/7/17 (IRB00092224). The study start date is 6/1/17 and estimated completion date is 5/31/20. The recruitment started in June 2017.

**Conclusions:**

This is a feasibility study that is meant to demonstrate the practicality of using fcMRI to study the neural correlates of PENFS outcomes and provide information regarding power calculations in order to design and execute a larger randomized controlled clinical trial to determine the efficacy of PENFS for improving pain and function.

**Trial Registration:**

ClinicalTrials.gov NCT03008837; https://clinicaltrials.gov/ct2/show/NCT03008837 (Archived by WebCite at http://www.webcitation.org/6wrY3NmaQ).

## Introduction

Fibromyalgia is a chronic pain syndrome that affects multiple body systems and is characterized by widespread pain, decreased physical function, fatigue, psycho-emotional and sleep disturbances and various somatic complaints [1,2]. It affects over 5 million Americans with an approximate female to male ratio of 7:1 [3]. A study derived from the US health insurance database found that the healthcare costs over 12 months are about three times higher among fibromyalgia patients when compared to patients without fibromyalgia. It is estimated that fibromyalgia costs the American population over 20 billion dollars per year in lost wages and disability [4]. This syndrome is not only devastating to the patient, but also represents a significant economic burden to the patient and society. Percutaneous electrical auricular stimulation of the vagus nerve has been utilized in the treatment of epilepsy and chronic pelvic pain [18,21]. In addition, we have previously reported a case of a female veteran with fibromyalgia who underwent PENFS with the NSS resulting in 100% relief of her fibromyalgia pain, Visual Analogue Scale (VAS) score from 8 to 0, reduction in opioid consumption and significant improvements in function for 4 months post-treatment, until the death of her father, at which point some pain symptoms began to return [22].

Fibromyalgia remains a poorly understood condition with regards to pathophysiology of the disease process, though maladaptive plasticity in the central (eg, brain) nervous system has been strongly implicated [5]. Current therapies are aimed at reducing the major symptoms of the disorder, such as treating the mood, sleep disturbances, and pain. These therapies include pharmacotherapy such as anticonvulsants and complementary and alternative medicine (CAM) therapies such as acupuncture. The current available therapies are often inadequate and frequently come with significant risks or side effects. These side effects often overlap with the symptoms of the disease, which result in poor patient outcomes. Some of the pharmacotherapy involved in treatment of fibromyalgia is aimed at addressing the pain symptoms. This often involves the utilization of opioids, possibly due to an exhaustion of other options for pain management. For pain conditions such as fibromyalgia, there is little evidence of benefit with chronic opioid therapy, and growing evidence against the use of opioids due to the risks for addiction, overdose, and side effects of long-term opioid use [6]. Given that opioid abuse is a major public health issue and drug overdose deaths are the leading cause of injury death in the United States, opioids should be avoided if possible [7]. With regards to CAM therapies, although these are often low risk and perceived to be beneficial by patients, there is not yet a sufficient level of evidence to support their use in fibromyalgia, limiting the ability of providers to make strong recommendations. More specifically, the combination of the small sample size, scarcity of studies for each comparison, and lack of an ideal placebo control weaken the level of evidence and the clinical implications of therapies such as acupuncture. Therefore, larger studies with higher levels of evidence are warranted [8].

Although the precise pathophysiology of fibromyalgia is not fully understood, there is a large consensus that it is a sympathetically or centrally mediated pain syndrome [9,10,11,12]. Comparisons have been made between fibromyalgia and complex regional pain syndrome (CRPS), a different sympathetically mediated pain syndrome. Fibromyalgia may be a widespread form of CRPS [13]. When treating sympathetically mediated pain syndromes, the goal of treatment is to interrupt the transmission of the sympathetic nervous system [13,14]. The vagus nerve is central to the parasympathetic autonomic nervous system, and modulation of its activity could result in effective treatment of sympathetically mediated pain through increasing parasympathetic outflow and modulating the sympathetic/parasympathetic balance [15]. Percutaneous electrical neural field stimulation (PENFS) via the ear is an intervention that aims to modulate the activity of cranial nerves (eg, vagus nerve at its auricular branches) and thus disrupt centrally mediated pain [16,17].

**Figure 1:**
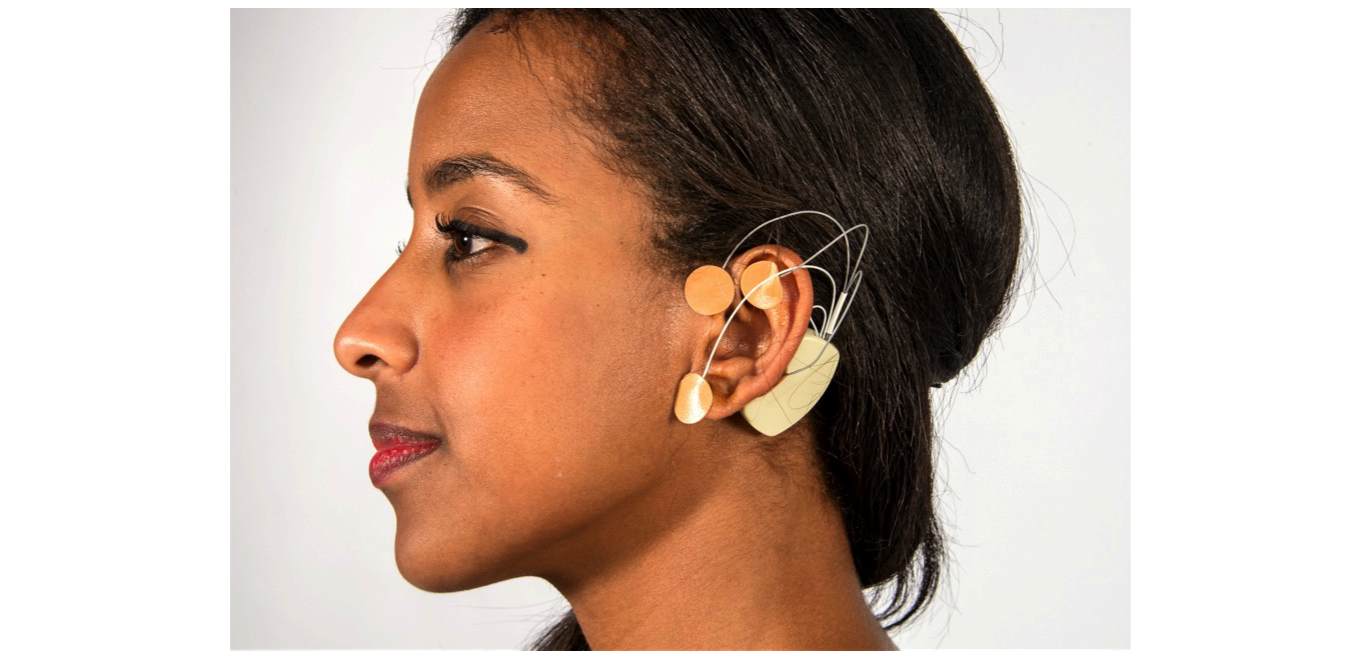
Military Field Stimulator/Neuro-Stim System.

Percutaneous electrical neural stimulation (PENS) involves the placement of needles near neurovascular bundles within a sclerodermal, myodermal or dermatomal distribution and delivering current to these structures [15]. The Military Field Stimulator/Neuro-Stim System (MFS/NSS) employs an evolved form of PENS known as PENFS (Figure 1).

PENFS of the auricle is similar to PENS, but instead of stimulating a certain neurovascular bundle it stimulates the entire ear and all the auricular branches of the cranial nerves, including the vagus nerve. The vagus nerve mediates sensation of the auricular tissue that makes up the ear; therefore, auricular stimulation has been used to modulate its activity and treat pain [16]. Napadow et al have demonstrated short-term relief of evoked pain sensation in chronic pain patients using electrical stimulation of the auricular branches of the vagus nerve [18]. PENFS is based on the idea that a central effect may occur through the creation of a field of electrical stimulation over peripheral branches of cranial nerves, and that this effect can be changed by varying the form, intensity and frequency of electrical current delivered to the neurovascular structures [19]. Animal studies have demonstrated that transcutaneous auricular stimulation of the vagus nerve can have antidepressant effects by stimulating the release of melatonin and serotonin in addition to significantly improving dental pain through the endogenous opioid system [20].

Although vagal nerve stimulation for pain relief has been studied, the mechanisms supporting PENFS stimulation to the auricle has not yet been studied with functional magnetic resonance imaging (fMRI) [23]. The basis for analyses of fMRI findings used in the present study is adapted from prior studies by Napadow and Harris et al using functional connectivity magnetic resonance imaging (fcMRI) to examine intrinsic brain connectivity in fibromyalgia patients before and after pharmacological and non-pharmacological therapy with pregabalin and acupuncture, respectively [24,25,26,27]. For example, their prior study demonstrated that fibromyalgia patients had significantly greater connectivity within the default mode network (DMN) and right executive attention network as compared to healthy, age-matched controls [25]. Additionally, greater connectivity was noted between the DMN and the insular cortex [25,26], a region of the brain implicated in evoked pain response and salience detection. Successful therapy was then found to reduce clinical pain and DMN-insula connectivity [27]. Gaining an understanding of the analgesic effects and neural correlates for PENFS therapy in fibromyalgia could result in (1) a better understanding of the pathophysiology of fibromyalgia, (2) cost-savings, (3) improvements in pain therapy, and (4) a decreased need for opioid analgesics.

## Methods

### Ethics Approval

This study attained approval by the Emory University/Veterans Affairs (VA) institutional review board and VA Research & Development committee. The study protocol will be conducted in accordance with ethical principles from the Declaration of Helsinki.

### Aims and Hypothesis

The specific aims of the study are (1) to evaluate the feasibility of using fcMRI as a biomarker for functionally correlated neural substrates of pain in patients undergoing PENFS and (2) to evaluate whether PENFS leads to analgesia and functional improvements as compared to standard treatment in veterans with fibromyalgia. Our primary hypothesis is that PENFS will result in decreased functional connectivity between the insula and default mode network as evaluated by fMRI [24,25,26,27], which will correlate to more significant improvements in pain and function relative to standard therapy for fibromyalgia.

### Design

This is a feasibility trial comparing PENFS to standard therapy in veterans with fibromyalgia. Enrollment according to the stated inclusion and exclusion criteria will be conducted and subjects who meet study criteria will undergo baseline assessments. These include a collection of bio-behavioral data such as cognitive and psychological assessments, eating, sleeping and drinking habits, and Patient-Reported Outcomes Measurement Information System (PROMIS) measures including physical function, anxiety, depression, fatigue, sleep disturbance, social function, pain interference, and global health. In addition, included are measures from the realms “Activity and Participation” from the International Classification of Functioning, Disability, and Health including arm curls, 30 second chair stands, and handgrip strength tests. Information from the Defense and Veterans Pain Rating Scale (DVPRS), and documented baseline analgesic consumption will also be included. Stratification based on age and sex will be performed to account for differential pain perception and neurological responses to pain based on age and gender. fMRI studies will then be conducted within 2 weeks prior to initiation of treatment and will be repeated within 2 weeks after the final treatment. Participants are advised to avoid caffeine and smoking the day of the neuroimaging analysis. The bio-behavioral assessments will be repeated at 4, 8, and 12 weeks after the final treatment.

**Figure 2:**
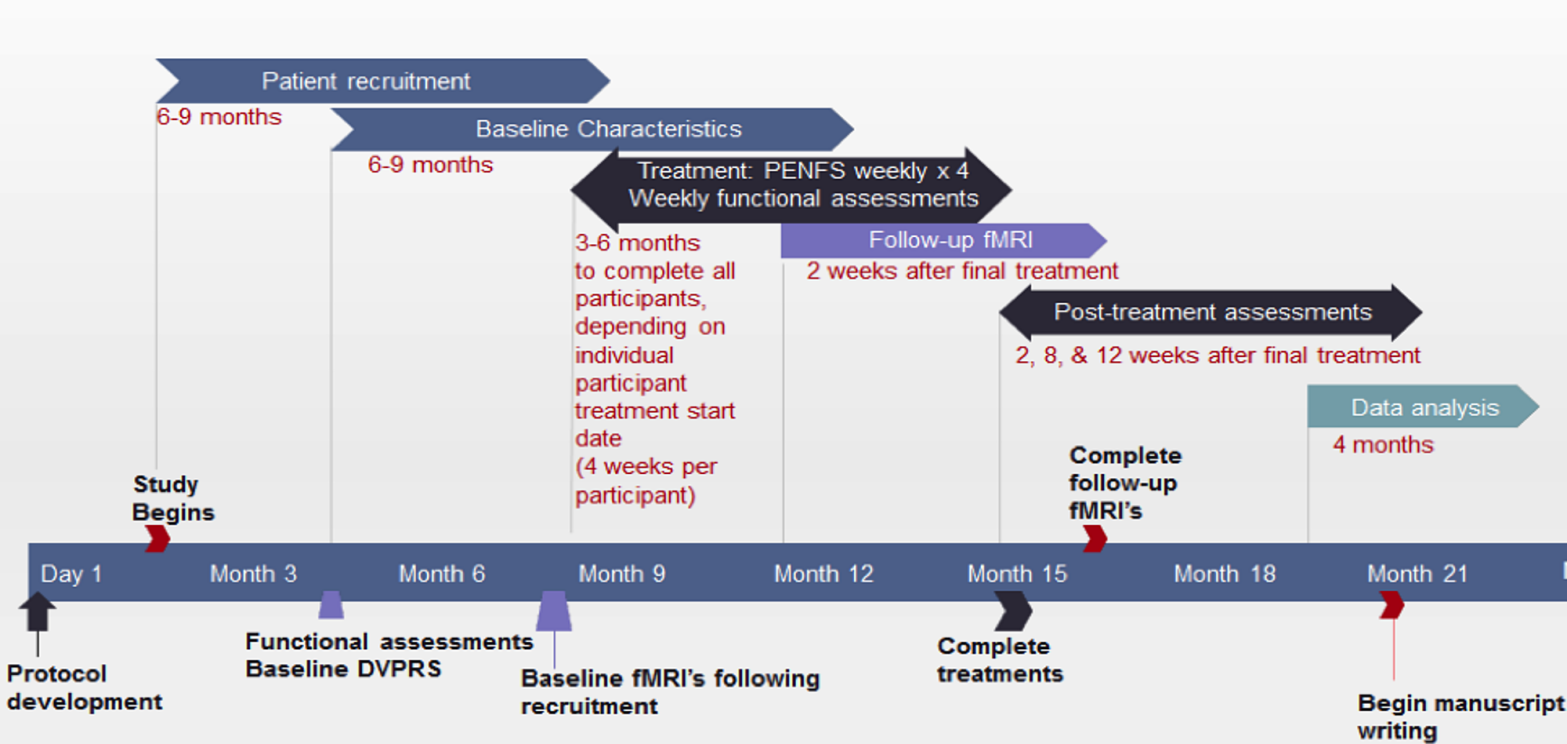
Anticipated timeline for study. DVRPS: Defense and Veterans Pain Rating Scale; fMRI: Functional magnetic resonance imaging; PENFS: Percutaneous electrical neural field stimulation.

The PENFS subject group will have the NSS placed on the ear for 5 days then removed and replaced at weekly intervals. The PENFS group will receive treatments once per week for a total of 4 weeks. Participants will be randomized to one of the two treatment groups. See Figure 2 for anticipated timeline for the study. Subjects will be block randomized, stratified by age and sex. This should provide an adequate sample size while minimizing confounding variables between groups. The standard therapy group will receive pharmacologic treatments such as anticonvulsants (ie, gabapentin, pregabalin), nonsteroidal anti-inflammatory medications (ie, ibuprofen, meloxicam), acetaminophen, topical agents and physical therapy individualized to patient comorbidities and preferences, as prescribed by a pain management practitioner, with regular check-ups at corresponding intervals to the NSS group.

### Setting

The fMRIs will occur at an Emory Imaging Center. All other visits will be conducted at the VA pain clinic in Atlanta, GA.

### Participants

Male and female Veterans age 20-60 with a diagnosis of fibromyalgia as diagnosed by a clinician, by chart review, and by the most recent American College of Rheumatology 2010 criteria for the diagnosis of fibromyalgia. The inclusion and exclusion criteria are listed in Textbox 1 [28,29].

### Randomization and Blinding

Subjects will be randomized to one of two groups; the control group will be standard therapy and the treatment group will be the PENFS treatment for this feasibility study. Simple randomization stratified by age and sex with equal allocation to treatment and control groups will be used. An age of 60 years old is set as a limit to minimize brain structural changes due to aging [30]. Subjects age 20-60, male and female with a diagnosis of fibromyalgia by the American College of Rheumatology 2010 criteria for the diagnosis of fibromyalgia will be included in the study [28,29]. Dr Kalangara, a pain physician trained to apply PENFS, will allocate subjects to one of the two treatment groups and will also perform the treatments. The principal investigator, Dr Woodbury, is a pain physician qualified to perform the pain and functional assessments and will perform these, blinded to treatment and control groups.

### Outcome Measures

The primary outcome measure is connectivity between the insula and DMN. Areas of the DMN found to be relevant in prior studies regarding fibromyalgia are the inferior parietal lobule (IPL), medial prefrontal cortex (MPFC), and posterior cingulate cortex (PCC) [25]. Based on existing data, fMRI seed-based resting connectivity analyses of the insula and relevant areas of the DMN (IPL, MPFC and PCC) will be performed [24,25,26,27]. Subjects will undergo fMRI studies within 2 weeks prior to initiation of treatment and will be repeated within 2 weeks after the final treatment for comparison.

**Inclusion and exclusion criteria.**

Inclusion Criteria:

- Subjects must be male and female Veterans age 20-60 with a diagnosis of fibromyalgia as diagnosed by a clinician, by chart review, and by the most recent American College of Rheumatology 2010 criteria for the diagnosis of fibromyalgia [28,29].
- Subjects must self-report consistent, daily pain (greater than 5 on a 0-10 VAS) >90 days.
- Subjects must have intact skin free of infection at the site of implantation.
- Subjects must be willing to participate and understand the consent.
- Subjects must be right-handed in order to provide consistency in brain structure and function.

Exclusion Criteria:

- Subjects must not be currently pregnant, since effects of fMRI and electrical current on the developing fetus are not well-known.
- Subjects must not have an implanted electrical device such as a vagal stimulator, pacemaker, or spinal pain pump, which are not compatible with MRI.
- Subjects must not have a history of seizures or neurologic condition that may alter the structure of the brain.
- Subjects must not have a history of drug abuse or severe, uncontrolled psychiatric illness such as schizophrenia or major depressive disorder with suicidal ideation.
- Subjects must not have psoriasis vulgaris or other skin conditions that may increase the risk of infection at the implantation site.
- Subjects must not have severe anxiety, claustrophobia, or other conditions that may prevent their ability to lie at rest in an MRI scanner. This will be determined after discussion with the patient regarding their own perceived ability to lie at rest in an MRI scanner without the use of additional sedating medications.
- Subjects must not introduce new medications or treatments for fibromyalgia symptoms during the course of the study, expect for those prescribed by the pain practitioners involved in the study, to prevent confounding results.
- Subjects must not have a concurrent autoimmune or inflammatory disease that causes pain such as systemic lupus erythematosus, inflammatory bowel disease or rheumatoid arthritis, since this could decrease the effect of treatment.
- Subjects must not experience trauma, injury or severe disease during the course of the study.

The secondary outcome is whether functional improvements occur with the application of PENFS, as the ultimate goal of reducing pain is to improve function. Secondary dependent variables for the evaluation of functional improvements with PENFS include PROMIS and International Classification of Functioning, Disability, and Health measures, the arm curl, 30 second chair stand and handgrip strength tests. These tests will be performed before treatment initiation and again at 4, 8 and 12 weeks follow-up after the completion of the 4-week treatment period for comparison.

### Sample Size

There will be a total of 20 subjects divided into 2 groups, 10 subjects in the control (standard therapy) group and 10 subjects in the PENFS treatment group. Age, gender, and comorbid conditions may confound effects on neurological response to pain, therefore subjects will be block randomized, stratified by age and sex. This will result in an adequate sample size while minimizing confounding variables between groups. Roberts and Brown demonstrated a decrease in VAS for pain using a series of 4 PENFS treatments in a cohort of 20 chronic pain patients [15]. Based on this Roberts and Brown study, we estimate the effect size to be a 4-point decrease in pain score, which is out of 10 points, in the PENFS group and a 2-point decrease in the standard therapy group.

Our study is a feasibility study being conducted with the purpose of providing better estimates of effect size and power calculations for sample size in future studies. The data gathered from this study will be used to inform power analyses for a future pilot study or randomized control trial. Therefore, we will have a power analysis after completing the study. 

### Recruitment

Recruitment will be performed through direct contact with patients at the pain clinic, and by letter invitation to veterans in the Atlanta VAMC system identified as carrying a diagnosis of fibromyalgia. Patients not already established in the pain clinic will only be contacted by study personnel with permission from the primary care physician.

There are sufficient numbers of patients at the Atlanta VAMC from which to recruit fibromyalgia patients, and due to the principal investigator’s role as a pain management physician, she has sufficient exposure to fibromyalgia patients within her own clinical practice. In order to demonstrate the feasibility of recruitment, we performed a data query for the Atlanta VA medical center, excluding satellite clinics, and found that over the course of a 1-year period (from March 26, 2014 to March 26, 2015), a total of 1,451 unique veterans were seen who carried a diagnosis of fibromyalgia. In age groups < 50 years old, there is a preponderance of females who carry the diagnosis, while in age groups > 50 years old, there is a preponderance of males. This is likely due to a gender difference in military enrollment in these age groups. The ratio of female to male veterans with fibromyalgia is more reflective of the general population in the <50-year-old age groups.  

On average, 2 patients are seen in the VA pain clinic with fibromyalgia per day, with 10 fibromyalgia patients a week. Of these, 60% would qualify for the present investigation. This is a conservative estimate. The prevalence of patients with fibromyalgia seen at the Atlanta VAMC and the pain clinic supports the feasibility of the study, given the planned recruitment of 20 study subjects from a population of at least 1,451 local veterans who carry the diagnosis of fibromyalgia, many of whom have been referred to and are followed in the pain clinic. We have allotted 6-9 months to recruit all patients. Given that 5 patients have already been recruited within 3 weeks of beginning the study, we don’t expect recruitment to be an issue.

### Statistical Analysis

In previous studies, the IPL, MPFC, and PCC are DMN regions that have been found to be relevant regarding fibromyalgia [26,27,28,29]. The primary outcome will be analyzed using fcMRI and analysis of functional neuroimages, a program that processes and displays fMRI data. The resting connectivity between the insula and DMN regions will be analyzed within 2 weeks prior to initiation of treatment to obtain a baseline and then again within 2 weeks after the final treatment. Data will be preprocessed and analyzed using the validated fMRI of the brain software library package and cardiorespiratory physiologic artifacts will be mitigated using retrospective image correction [31,32,33]. Further, artifacts related to subject motion will be minimized in fcMRI time series using the validated independent component analysis (ICA) based automatic removal of motion artifacts tool. This algorithm is a data-driven method to identify and reduce motion-related artifacts (ICA components) from fMRI data. Given that drifts in MRI acquisition are typically considered linear, first order polynomial fitting will be utilized to account for MRI signal drift.

The primary outcome measure is connectivity between the insula and DMN as a biomarker for pain. Based on previous research, we hypothesize that the addition of PENFS will decrease connectivity between the insula and DMN structures relative to standard treatment [24,25,26,27]. Z-scores from two correlations described above (ie, between the posterior insula and the IPL and between the posterior insula and the PCC) will be used as the dependent variables for this outcome analysis. Hence, changes in z-scores for the two correlations will be tested for each group using pairwise comparisons. We expect that posttreatment connectivity between the posterior insula and DMN structures will be reduced from pre- to post-PENFS treatment. We do not expect to see significant changes for standard therapy at 2 weeks following the final treatment. Each of the two z-transformed correlations, for both pre- and post-PENFS and pre- and post-standard therapy, will be family-wise error (FWE) corrected (voxel-wise, and cluster-wise) to *P*<.010, and cluster size of 20 for the two correlations for each group. Patient-reported changes in pain will be evaluated using (1) DVPRS severity scores and (2) analgesic consumption before and after treatment (4 weeks) and at long-term follow-up (8 weeks, 12 weeks) following the 4th week of treatment for each group. We will use 2 groups (PENFS versus control) 4 times (pre- and post- treatment at 4, 8, and 12 weeks follow-up) for repeated measures analysis of variance to compare outcome for the groups over time. We will employ a generalized linear mixed model (GLMM) framework to fit the ANOVA model to the data. GLMM approach is more robust than traditional ANOVA methods as it better handles the possibility for imbalance in the effects due to dropout and other losses to follow-up and provides a stronger estimate of the fixed group effect while controlling for time as a random effect in the model. After the completion of the above analyses, we will use Spearman Rank correlation coefficients to investigate the predictive ability of pre-PENFS (baseline) resting insula connectivity to predict post-PENFS changes in pain levels. Baseline connectivity will be extracted as z-scores from imaging data. Categorical variables such as gender and bio-behavioral data will be assessed using Fisher’s exact test, but continuous variables such as age will be assessed using two-tailed t-tests. All reported *P* values will be 2-tailed and considered significant at the .05 level, FWE corrected.

We will also perform a linear regression with z-scores from fcMRI correlations and baseline pain levels using the DVPRS in order to evaluate links between baseline resting insula connectivity and baseline individual differences in pain sensitivity. In addition, we will also use a linear regression model to assess any association between treatment-modulated clinical pain and changes in the posterior insula resting brain connectivity.

The secondary outcome is whether functional improvements occur with the application of PENFS, as the ultimate goal of reducing pain is to improve function. Secondary dependent variables for the evaluation of functional improvements with PENFS include PROMIS and International Classification of Functioning, Disability, and Health measures, the arm curl, 30 second chair stand and handgrip strength tests at 4, 8, and 12 weeks follow-up after the completion of the 4-week treatment period. Pairwise repeated measures comparisons between pretreatment and posttreatment DVPRS, analgesic consumption and functional assessments will be performed within each group, FWE corrected to *P*<.050. Similar analyses will be conducted at 6 and 12 weeks follow-up. Data from each time point can be considered its own family of comparisons for this purpose. Further, analysis of sample characteristics for the groups, PENFS versus control, will be conducted to assess comparability of the samples. Categorical variables such as gender and bio-behavioral data will be assessed using Fisher’s exact test, but continuous variables such as age will be assessed using two-tailed t-tests. All reported *P* values will be 2-tailed and considered significant at the .05 level, FWE corrected. Data collected and analyzed regarding functional changes related to PENFS treatment will be assessed for new hypothesis generation.

## Results

This trial has been funded by the Veterans Health Administration Program Office. This study attained approval by the Emory University/VA institutional review board and VA Research & Development committee. Institutional review board expedited approval was granted on 2/7/17 (IRB00092224). The study start date is 6/1/17 and estimated completion date is 5/31/20. The recruitment started in June 2017.

## Discussion

Fibromyalgia is a syndrome that, despite affecting millions of Americans, remains a difficult condition to treat. The current therapies continue to fall short and many times leave these patients with intolerable side effects. PENFS is a Food and Drug Administration approved, non-pharmacologic therapy that is currently utilized within the military and VA system, but sufficient evidence regarding its outcomes and neural mechanisms have not been adequately investigated. Auricular PENFS has not been studied with fMRI. Stimulation of the auricle may produce neural changes that differ from traditional therapies. Understanding the underlying neural mechanisms of auricular PENFS could assist in developing targeted treatments for fibromyalgia and chronic pain. An understanding of its neural underpinnings and analgesic effects could lead to improvements in pain management and quality of life, cost-savings, and development of new techniques to address pain.

The present investigation is a feasibility study being conducted with the purpose of providing better estimates of effect size and power calculations for sample size in future studies. The data gathered from this study will be used to inform power analyses for a future pilot study or randomized control trial. This study will not only serve to elucidate neural changes with PENFS, but could provide evidence regarding the relative effectiveness of this already clinically employed non-pharmacologic treatment. This in turn can result in evidence-based implementation that be utilized to treat not only veterans suffering from fibromyalgia but also all fibromyalgia patients. Informed consent will be obtained from all trial participants.

## Appendix

Multimedia Appendix 1 Original NIH peer-review reports.

## Abbreviations

CAM: complementary and alternative medicine
CRPS: complex regional pain syndrome
DMN: default mode network
DVPRS: Defense and Veterans Pain Rating Scale
fcMRI: functional connectivity magnetic resonance imaging
fMRI: functional magnetic resonance imaging
GLMM: generalized linear mixed model
ICA: independent component analysis
IPL: inferior parietal lobule
MPFC: medial prefrontal cortex
MFS/NSS: Military Field Stimulator/Neuro-Stim System
PROMIS: Patient-Reported Outcomes Measurement Information System
PENFS: percutaneous electrical neural field stimulation
PENS: percutaneous electrical neural stimulation
PCC: posterior cingulate cortex
VA: Veterans Affairs
VAMC: Veterans Affair Medical Center
VHA: Veterans Health Administration
VAS: Visual Analogue Scale
