# Supplementary material for: Functional Magnetic Resonance Imaging Evaluation of Auricular Percutaneous Electrical Neural Field Stimulation for Fibromyalgia: Protocol for a Feasibility Study
Source: JMIR Res Protoc. 2018 Feb 6;7(2):e39. doi: 10.2196/resprot.8692 (PMC5820456; doi:10.2196/resprot.8692)
Supplement: Multimedia Appendix 1 [file resprot_v7i2e39_app1.pdf]

SUMMARY STATEMENT
(Privileged Communication)

PROGRAM CONTACT:
PhD Brian Schulz PHD
(202) 443-5769
brian.schulz@va.gov

Release Date: **09/02/2016**
Revised Date:

Application Number: **1 IK1 RX002113-01A2**

Principal Investigator

WOODBURY, ANNA

Applicant Organization: VETERANS HEALTH ADMINISTRATION

Review Group: **RRD9**
Career Development Program - Panel II

Meeting Date: **08/03/2016**
Council: **OCT 2016**
Requested Start: **11/01/2016**
RFA/PA: **RX16-017**
PCC: **RXSCHU**

Project Title: **Feasibility Study: fMRI Evaluation of Auricular PENFS for Fibromyalgia**

SRG Action: **Impact Score:145**

Human Subjects: **20-Human subjects involved - No exemption designated**
Animal Subjects: **10-No live vertebrate animals involved for competing appl.**
Gender: **4A-Gender representation unknown, scientifically acceptable**
Minority: **4A-Minority representation unknown, scientifically acceptable**

Project Year	Direct Costs Requested
1	234,966
2	234,966
TOTAL	469,932

SUMMARY OF DISCUSSION:
The Board met in Plenary Session and reviewed the above proposal considering all internal and external reviews. This document summarizes the major points of the discussion concerning the proposed project. In any further development of this project, the investigator should consider carefully all the issues reflected in this Summary of Discussion as well as the more detailed comments in the individual critiques.

GENERAL COMMENTS:
The panel discussed the second resubmission of this CDA-1 from Dr. Woodbury. In general the panel was enthusiastic about the applicant, who is an acting VA physician and licensed acupuncturist, and is interested in building evidence in pain management treatments for people with fibromyalgia. The panel agreed that this work is significant and the applicant was generally responsive to the prior critique. There was some discussion regarding whether all members of the mentor team were fully invested given that the new clinical trials mentor states he can only devote 1% of time on mentorship and wording around addition of requested measures on activity and participation seemed to confuse PROMIS (as measures) and the ICH (as a framework). The project appears feasible to execute and responsive to previous critiques although some concern was raised about the use of a low sample size in the trial. Ten participants per group may not be large enough to generate a publication that would bolster a future CDA-2 application. One suggestion to consider is the use of a crossover design. Despite some weaknesses, the application concerns a significant research area from a promising candidate who has great potential to become an independent VA research investigator. There was some skepticism that fcMRI could truly provide an “objective measure” of pain, as pain is generally understood to be a subjective experience, informed by multiple domains of physical, physiological, and psychosocial factors. However it is recognized that fcMRI might be useful in illuminating underlying neural mechanisms related to pain.

DESCRIPTION (provided by applicant):
BACKGROUND: Fibromyalgia is a chronic pain syndrome that consists of chronic widespread pain, decreased physical function, fatigue, pyschoemotional/sleep disturbances, and various somatic complaints, affecting anywhere from 5-10 million Americans, with ~1,500 Veterans carrying a diagnosis of fibromyalgia seen per year at the Atlanta VAMC alone. It is estimated that fibromyalgia costs the American population over $20 billion/year in lost wages and disability. Initial therapies often include CAM therapies, which are generally considered safe, although their efficacy has not been thoroughly evaluated for fibromyalgia. Thus, non- pharmacologic alternatives require more rigorous scientific investigation for the treatment of fibromyalgia. There is evidence to support the use of percutaneous electrical neural stimulation (PENS) in the treatment of pain conditions, which may have increased effects relative to acupuncture based on systematic reviews. While fMRI data for acupuncture and fibromyalgia exists, no such data exists for PENS treatment. An evolved form of PENS, percutaneous electrical neural field stimulation (PENFS) of the auricle is already used in the military and VA systems for the treatment of chronic pain, but evidence regarding its mechanisms and effects is lacking. [Stimulation of the vagus nerve, which has auricular branches, has been previously studied for pain relief in fibromyalgia. However, application of PENS-type stimulation to the auricle has not been previously studied with fMRI, and this type of therapy may lead to neural changes worthy of further exploration.] OBJECTIVE: To evaluate the feasibility of using fMRI as a biomarker for the neural substrates of pain and functional changes following PENFS treatments. HYPOTHESIS: PENFS will result in decreased functional connectivity between the insula and default mode network as evaluated by fMRI, which will correlate to more significant improvements in pain and function relative to standard therapy for fibromyalgia. METHODS: Subjects who meet study criteria will receive baseline assessments including resting state fMRI, collection of biobehavioural information such as cognitive and psychological assessments on standardized forms, eating, sleeping and drinking habits, Defense and Veterans Pain Rating Scale (DVPRS), [PROMIS measures], arm curl, 30-s chair stand, and handgrip strength tests, [measures from the realms “Activity and Participation” from the International Classification of Functioning, Disability, and Health,] and baseline analgesic consumption. [Subjects will

be stratified based on age and gender and then divided into standard therapy control (medication management and physical therapy)] or PENFS (series of 4, weekly) treatments and assessed for fMRI changes 2 weeks after the final treatment. [Pain and function will also be assessed at the 2 weeks, 6 weeks and 12 weeks] following the final treatment. CLINICAL RELEVANCE: [Auricular PENFS has not been studied with fMRI. Stimulation of the auricle may produce neural changes that differ from traditional therapies. Understanding the underlying neural mechanisms of auricular PENFS could assist in developing targeted treatments for fibromyalgia and chronic pain.] Further, if PENFS can significantly improve pain relief and function over standard therapies, it could decrease the need for opioid analgesics and their associated risks, which is a primary objective of the VA Opioid Safety Initiative. The present investigation will not only serve to elucidate neural changes with PENFS, but could be directly applicable to our Veterans suffering from fibromyalgia by providing evidence regarding the relative effectiveness of this already clinically-employed non- pharmacologic treatment (or lack thereof), and result in evidence-based implementation and potential cost savings to the VA system.

PUBLIC HEALTH RELEVANCE
Given recent increasing opioid-related deaths and evidence showing against the use of opioids for non-malignant chronic pain, there is growing need for non-narcotic pain management. Fibromyalgia is a difficult to treat chronic pain condition that is often treated with opioids despite existing evidence. The prevalence of fibromyalgia is increased among Veterans returning from the gulf war and is already a significant burden in senior Veterans who may have suffered with chronic pain for decades already. Many treatment options for fibromyalgia carry intolerable side effects. PENFS (percutaneous electrical neural field stimulation) is a FDA- approved, non-pharmacologic therapy that is currently utilized within the military and VA system, but sufficient evidence regarding its outcomes and neural mechanisms have not been adequately investigated. An understanding of its neural underpinnings and analgesic effects could lead to 1) improvements in pain management and quality of life, 2) cost-savings and 3) development of new techniques to address pain.

CRITIQUE 1

DESCRIPTION: This is a second resubmission of a CDA-1 grant in which the goal is to examine the feasibility of using functional connectivity MRI (fcMRI) as a biomarker to investigate functionally correlated neural substrates of pain in patients with fibromyalgia who are undergoing auricular percutaneous electrical neural field stimulation (PENFS) for pain reduction and to evaluate whether PENFS leads to improvements in pain and function compared to standard treatment in Veterans with fibromyalgia.

CRITIQUE (Applicant, Mentor, and Training Program):

Applicant: The candidate is a licensed acupuncturist and board certified pain management physician with a special interest in fibromyalgia. She is currently the Division Chief for pain management at the Atlanta VAMC, and uses the two forms of pain treatment PENS and acupuncture in treatment in the management of chronic pain on a regular basis. She is also a faculty member at Emory University where some of the study procedures will take place. She currently has 12 publications, most recently a narrative review on CAM therapies for pain practitioners. She appears highly committed to building evidence around the use of minimally invasive integrative therapies, like acupuncture and PENFS, for use in her patients as well as to understand underpinnings of chronic pain in fibromyalgia.

Mentor/s: There have been changes to the mentor team in response to previous review critiques. Her primary mentor is Dr. Crossen who is the Executive Associate Director in Center for Visual and Neurocognitive Rehabilitation (CVNR) at the Atlanta VAMC and also directs the neuroimaging core. He has a good history of mentoring junior faculty and will teach her fMRI as it relates to this project which is a central skill she hopes to acquire. Dr. Sniecinski is an anesthesiologist at Emory University, has

performed clinical research studies in the field, and is charged with teaching her more practical aspects of clinical trial implementation and learning appropriate statistical analyses. Dr. Napadow is an external mentor from the Boston area who is known for his content expertise in acupuncture and pain management. He is tasked with providing monthly readings and discussion regarding the assessment of acupuncture with fMRI and with helping her with grant writing. He will be an essential mentor in the field of functional connectivity MRI which he pioneered and will be employed in this study. Reviewers requested the addition of a mentor who had particular expertise in clinical trials and now Dr. Lawrence Phillips has been added to the team. He is the Director of the Clinical Studies Center at the Atlanta VAMC as well as a faculty member at Emory in the medicine and physiology departments. Dr. Phillips was a site PI on the Women's Health Initiative, had several R01 grants and is program director of an NIH training grant that has been renewed for the past 20 years. He has extensive experience in training faculty members and fellows in clinical research. Dr. Kalangara, a pain physician who works with Dr. Woodbury, will serve as a collaborator in the project, will place the auricular PENFS devices on patients to maintain blinding of Dr. Woodbury, and will serve as Acting Chief during her dedicated research time. Dr. Garcia and Dr. Huang are two other consultants with expertise in mechanisms of neuromodulation and developing acupuncture protocols respectively. Dr. Guo is a biostatistician who will consult with Dr. Woodbury about the independent component analysis, a method which was suggested by reviewers in the last review. Dr. Krishnamurthy works in Dr. Crossen's neuroimaging core and will also consult on the research project.

All of these team members together depict a strong network of support and mentorship from a variety of sources. The addition of Dr. Phillips was important to round out mentorship in clinical trial design and conduct. In future applications, it would be nice to have letters from all of these people. The letters section had current letters of most senior key personnel (except Dr. Sniecinski) and old letters from previous applications from people no longer on the team (such as Dr. Mackenzie-Brown).

Training Program: The candidate's goals for the CDA-1 are 1) to develop a foundation of knowledge in important tools for neuroimaging for the objective evaluation of pain and analgesia such as fMRI, 2) to develop experience in conducting a clinical trial, and 3) to become well versed in pain and acupuncture research. Her training plan involves targeted mentorship with each of her mentors and to undertake the Masters of Science in Clinical Research Program at Emory. Her coursework will cover neuroimaging and clinical trials development as well as biostatistics and appears to be carefully thought out to address her goals.

CRITIQUE (Scientific Merit—Strengths, Weaknesses, & Recommendations):

Significance: This project is significant for several reasons. It provides evidence in minimally invasive pain treatment for a difficult-to-treat population of people with fibromyalgia, who often receive opioid treatments. Neurostimulation such as PENS is currently being used in Veteran populations and could be feasibly applied. The use of functional connectivity MRI provides an important opportunity for Dr. Woodbury to learn from Dr. Napadow and other mentors in neuroimaging as well as extend the study of the use of fcMRI as a biomarker in fibromyalgia.

Approach: Dr. Woodbury and team have structured the aims to replicate and extend similar research that has been done by a member of her mentor team and others that has established brain connectivity biomarkers to examine acupuncture effects in patients with fibromyalgia.

Data presented in the Preliminary data and feasibility section should not include studies from other researchers (e.g. Roberts and Brown study) that are not part of the team. Data from previous studies in the larger body of knowledge belong in the Background and Significance and is otherwise potentially misleading.

Environment: The environment is strong. The Atlanta VA has an RR&D Center of Excellence in Visual and Neurocognitive rehabilitation. This center has a neuroimaging core which has been utilized to develop methodology for the data analysis as reported by the candidate. In addition, there are new resources being used at Emory with the addition of Dr. Phillips to the team.

Feasibility: The applicant responded well to critiques about the feasibility of the project in this submission. Given the mentorship in place and the discussion of the clinical trial and the fcMRI aims, the feasibility appears strong.

Overall Evaluation (Applicant/Mentor(s)/Training and Scientific Merit):
The applicant responded well to the prior critiques and appears well positioned to pursue this CDA-1. While grant and scientific writing is addressed in the training plan, the applicant should work to publish papers related to topics in the project that are data-driven with her mentors.

Ethical/Safety Issues: None noted.

OTHER CONSIDERATIONS:

Clinical Relevance: This work is highly clinically relevant, as it will help identify whether PENFS may improve pain and functional outcomes in this sample which can lead to larger studies in the area.

Budget: Travel to Dr. Napadow's clinic was reduced and other items questioned in the last critique such as supplies have now been justified.

CRITIQUE 2

DESCRIPTION: Fibromyalgia affects 5-10 million Americans, with 1,451 Veterans carrying a clinical diagnosis of fibromyalgia annually at the Atlanta VAMC alone according to a recent data query. Though this syndrome more often affects females, due to the higher male to female ratio in the Veteran population both gender groups are well represented with this disease. Pain related to fibromyalgia is often difficult to manage and results in increased utilization of healthcare services, lost workdays, and overall disability. A safe and effective treatment for fibromyalgia is needed.

Percutaneous application of electrical neural stimulation (PENS) and auricular PENS have provided evidence for analgesia in clinical trials. The Military Field Stimulator (MFS/Neuro-Stim System), a percutaneous electrical neural field stimulation (PENFS) device evolved from PENS, is currently employed by the United States (US) military and used in the VA (contract number V797D-50453). There is some evidence based on a small trial of 20 postoperative patients that suggests PENS may have greater benefit than acupuncture for acute pain. However, while the effects of acupuncture treatment have been explored with fMRI and correlated with analgesic improvements, such data do not exist for auricular PENS/PENFS. In fibromyalgia patients, decreased intrinsic brain connectivity on fMRI has been correlated with a reduction in pain scores, and analgesic interventions have decreased connectivity between the default mode network (DMN) and various regions of the insula, normalizing towards healthy controls. OBJECTIVE: This investigation intends to examine the feasibility of using resting state functional connectivity MRI (fcMRI) as a biomarker for PENFS-induced changes in the treatment of fibromyalgia. Auricular PENFS shares similarities with auricular acupuncture (in that it utilizes the ear) and PENS (in that it utilizes percutaneous electrical stimulation). While these therapies are currently clinically employed, only acupuncture (not involving the ear) has been studied in fibromyalgia patients using fMRI. Thus, there is a premise for using fMRI as a biomarker in chronic pain patients, but it is possible that the application of stimulation specifically to the ear may result in differential neural effects as compared to acupuncture in fibromyalgia patients.

The candidate's goals are: 1) to develop a foundation of knowledge in important tools for neuro-imaging for the objective evaluation of pain and analgesia such as fMRI, which will be of use for subsequent applications, 2) to develop experience in conducting a clinical trial and 3) to become well versed in pain research for nonpharmacologic therapies.

CRITIQUE (Applicant, Mentor, and Training Program):

Applicant: The candidate has broad experience, interest and training in acupuncture and in allopathic medicine (anesthesiology). She has experienced some basic research training through a Foundation for Anesthesia Education and Research (FAER) fellowship, and advanced clinical training through a pain management fellowship in anesthesiology at Emory University. She is now the lead physician for pain management at the Veterans Affairs Medical Center in Atlanta, GA, treating chronic pain with a combination of mainstream and alternative medical techniques ranging from conservative management with heat, ice, and stretching to pharmacological intervention to acupuncture to minimally invasive, fluoroscopically guided, interventional techniques. She is in a good position to recruit participants. Additionally, she is on the Committee on Pain Medicine for the American Society of Anesthesiologists. Although she has some publications, she lacks experience in clinical trials and fMRI. It is expected that the applicant would gain the necessary experience and knowledge through the CDA process.

Mentor/s: Dr. Woodbury is supported by an accomplished mentorship team, led by Dr. Crosson, a well-regarded researcher in the areas of fMRI, neuroimaging and cognitive function. He is currently the primary mentor of a PhD (RR&D CDA-2) and is a secondary mentor for four others. He [will assure that Dr. Woodbury gets assistance in running the clinical trial.] Vitaly Napadow will add his experience in the particular area of functional neuroimaging as it relates to pain and fibromyalgia. Dr. Sniecinski will help with some of the practical aspects of mounting a study such as organization and compliance with IRB requirements. Dr. Lawrence Phillips, MD, Director of the Clinical Studies Center at the Atlanta VAMC has agreed to be a clinical trial design mentor for the applicant and will devote 1% of his time to this. Wei Huang, MD, PhD, is a consultant who physiatrist/acupuncturist, who may provide some rehabilitation perspective to the process.

Training Program: The training program a Master's of Science in Clinical Research degree program from the Atlanta Clinical & Translational Science Institute in the second year. One expects that the candidate would be accepted into the program, although there is not a contingency plan if she is not. The trips to Dr. Napadow's lab have been reduced, which should make the experience more feasible. The plan could be strengthened with the addition of education in behavioral approaches (exercise, cognitive behavioral therapy) to pain/fibromyalgia.

CRITIQUE (Scientific Merit—Strengths, Weaknesses, & Recommendations):

Significance: Fibromyalgia and related chronic pain syndromes are common, difficult to manage, and not well understood. They cause misery to affected individuals and vex providers. fMRI is one tool to help gain understanding of the neuroanotomic mechanisms underlying the conditions and such insights neurostimulation such as PENFS, are important, and as they hold the promise of symptom relief free of many of the deleterious side effects associated with medicines. This trial would be similar to others of Dr. Napadow studying the intervention of acupuncture, with the major difference being that instead of acupuncture, the intervention would be PENFS. The candidate plans future trials comparing PENFS to battlefield acupuncture, which is important as battlefield acupuncture is likely to rely a similar mechanism of action and is significantly less expensive, and gaining traction as a practice in the VA and DoD.

Approach: The applicant has been responsive to the major concerns of the reviewers. The scope of the trial has been reduced to a more doable feasibility trial. Twenty Veterans with fibromyalgia will be randomized into either a treatment (four weeks of PENFS) or a control group (four weeks of usual

standard of care treatment with the restriction that no new treatments be introduced). The control group will be block randomized, stratified for age, gender, although not by comorbidities. The PENFS units are still being purchased as a clinical item. Although they are being used in a fashion consistent with their manufacture, they are not being used as a treatment of last resort for pain as is the common practice at the Atlanta VA, but as part of a research protocol. This amounts to a donation to the CDA from the clinical prosthetics service. Dr. Kalangara, will perform the interventions, allowing the PI to perform functional assessments and data analyses in a blinded fashion. The trial now closely matches the form of trials of her mentor, Dr. Napadow, which is consistent with the format of a CDA-1. (Napadow, V.; Kim, J.; Clauw, D. J.; Harris, R. E. Decreased intrinsic brain connectivity is associated with reduced clinical pain in fibromyalgia. Arthritis Rheum 2012, 64, 2398-2403). The imaging portion of the trial now appears strengthened with the addition of independent component analysis and the inclusion of explicit head movement reduction and correction protocols. The inclusion of PROMIS measures should strengthen the application. The ICF is a conceptual model, not a measure in and of itself, which seems to have been missed by the applicant and team. There is a crosswalk between the ICF and the PROMIS measures. The goal of developing an objective measure of pain could use further explanation. While it is easy to accept that imaging techniques can elucidate the physiologic mechanisms of sensations, the experience of pain seems by its nature to be subjective and dependent on multiple factors including the physiologic experience and the context of the experience, as well as the motivations and goals of the individual. It appears that participants are to be excluded if they experience adverse events such as vasovagal syncope, skin irritation, or they remove the device. Assumedly, they will have already enrolled in the study and have gone through the informed consent process. Even if they aren’t able to finish the protocol, they should be included in the study.

Environment: The environment appears sufficiently strong to support the candidate. The Atlanta VA RR&D Center of Excellence (CoE) includes sufficient office space, laboratories, data support, and mentorship to support the candidate and the project. The candidate’s position in the Atlanta Clinic and Atlanta VA Medical Center- Pain Management and Acupuncture Facilities, should present an adequate population of Veterans for recruitment. The Center for Systems Imaging (CSI) and the Neuroimaging Analysis Laboratory are available to Dr. Woodbury to conduct the scanning and analysis as proposed.

Feasibility: The project appears feasible. The proper equipment, mentoring and institutional support is in place. An adequate population should be available. A timeline for the study has been added, which is helpful. The candidate is qualified and motivated, and has assembled a strong team of mentors and reasonable education plan, and has identified an important area in need of investigation. A plan for reduction of clinical responsibilities has been included, and the amount of travel has been reduced.

Overall Evaluation (Applicant/Mentor(s)/Training and Scientific Merit):
The candidate is accomplished, qualified and motivated, and has assembled a strong team of mentors and a reasonable educational plan, and has identified an important area in need of investigation. The applicant has been responsive to the major points of concern of the reviewers, and as a result, the research plan is more realistic and more feasible, and less ambitious. The current design should produce the data necessary to compute the effect size of the intervention, and lay the groundwork for larger, more definitive trials. The applicant/team seems confused about the ICF model and how it interfaces with the PROMIS measures. The goal of establishing an objective measure of pain could use some clarification. Participants who are withdrawn from the trial after consenting should be included in the study, particularly if the withdrawal has to do with adverse events.

Ethical/Safety Issues: No new issues.

OTHER CONSIDERATIONS:

Clinical Relevance: The potential role of acupuncture (including battlefield acupuncture) and neurostimulation in the treatment of fibromyalgia and pain in general are important to the VA and to the

public at large. Rigorous research that helps to establish the effects, limitations, and mechanisms of such approaches are needed to help Veterans and others. This line of work has the potential to help integrate needed treatments more broadly amongst the medical community.

Budget: The budget of the CDA-1 is restricted to exclusively to support the applicant's salary. Running clinical trials is a large undertaking. It is good to know that the RR&D CVNR has staff available to assist. An actual statement of commitment from the RR&D CVNR to support the study with the personnel to assist the conduct of the trial would be even more impressive.

Other Issues:
- Data and safety monitoring plan is adequate.
- Inclusion of women and minorities: No specific plans to recruit women and minorities.
- However, the sample is relatively small, so that representation by of minorities may not be an issue.
- Assumedly, the Atlanta VAMC has a large population of African-American Veterans. Since fibromyalgia disproportionately affects women, it should be relatively easy to recruit women.

CRITIQUE 3

DESCRIPTION: No comments.

CRITIQUE (Applicant, Mentor, and Training Program):

Applicant:
Strengths:
The applicant has a history of research and publications in pain and alternative therapies. Her career goals and focus align with the mission of the VA. As a VA clinician, she has useful insight to the practice of pain management in Veterans.

Weaknesses:
- No publications with primary mentor.
- The applicant seemingly has no experience even as a collaborator on neuroimaging publications.

Mentor/s:
Strengths:
The addition of a clinical trials mentor has strengthened the mentorship team.

Weaknesses:
None noted.

Training Program:
Strengths:
The formal coursework in fMRI and training by collaborators with neuroimaging experience is strong.

Weaknesses:
- Simultaneous enrollment in year 2 in the Master's program, fMRI coursework, and conducting research activities is overly ambitious.
- The availability of local (CVNR) resources for hands on neuroimaging analysis and data management could be expanded.

CRITIQUE (Scientific Merit—Strengths, Weaknesses, & Recommendations):

Significance:

Strengths:
- The proposal addresses a significant issue in pain management and proposes a widely available intervention for an important issue in Veterans' health.
- The reduction of pain and secondary effects of pain management (opiod use), will provide a considerable benefit to the quality-of-life and financial burden if the treatment proves to be effective.

Weaknesses:
The role of neuroimaging in pain management, while interesting from a scientific standpoint, has an unclear path to clinical adoption and personalized medicine.

Approach:
Strengths:
The potential pitfalls are well thought out.

Weaknesses:
Even for a feasibility study, 10 subjects per group are low for fMRI analysis. Employing more subjects or a crossover design in all 20 subjects could increase the power (Ref 18, for instance). How will placebo effects of the PENFS group be considered?

Environment:
Strengths:
The environment is strong. The VA Pain Clinic and the CVNR Neuroimaging Core provide access to necessary patient and MRI facilities, respectively.

Weaknesses:
None noted.

Feasibility:
Strengths:
- The utilization of PENFS devices already in practice facilitates their adoption to this study.
- The support of neuroimaging fees through the center is an advantage.

Weaknesses:
Additional preliminary evidence in the use of PENFS, even in CRPS subjects from their VA Pain Clinic, would support the experience of using the device. How many times has the device been administered? What is the level of compliance in these patients?

Overall Evaluation (Applicant/Mentor(s)/Training and Scientific Merit):
This is a resubmission of a prior application for CDA-1. Overall, the applicant background is strong and has a history and expertise in clinical pain management setting. The mentorship team is diverse and also has expertise in the research and mentoring activities. The training plan to incorporate neuroimaging tools into pain management research is well formulated, but still ambitious.

The proposal has high significance to clinical practice and a particular relevance to the mission of the VA. The experimental design could be strengthened by additional preliminary data regarding the feasibility of using the PENFS device in practice. The enrollment for the fMRI studies leaves it underpowered even without considering patient non-compliance and dropout rates, which are not given.

Ethical/Safety Issues: None noted.

OTHER CONSIDERATIONS:

Clinical Relevance: While fMRI has shown considerable promise in monitoring pain and the brain mechanisms of pain in group studies, its clinical utility for individual patient monitoring or as a predictive biomarker is limited. How will the results of this study further the goal of using fMRI for individual patients?

Budget: The budget is appropriate.

MEETING ROSTER
Career Development Program - Panel II
Rehabilitation Research and Development Parent IRG
Office of Research & Development

RRD9
08/03/2016

CHAIRPERSON(S)

WECHT, JILL M., EDD
RESEARCH ASSOCIATE/PRINCIPAL INVESTIGATOR
SPINAL CORD DAMAGE RESEARCH CENTER
JAMES J. PETERS VA MEDICAL CENTER (BRONX)
PROFESSOR OF MEDICINE AND REHABILITATION MEDICINE
MOUNT SINAI SCHOOL OF MEDICINE
BRONX, NY 10468

MEMBERS

ACIERNO, RONALD E., PHD *
PROFESSOR
PTSD CLINICAL TEAM
CHARLESTON VAMC
PROFESSOR, DEPT OF PSYCHIATRY AND BEHAVIORAL SCI
MEDICAL UNIVERSITY OF SOUTH CAROLINA
CHARLESTON, SC 29403

BEGUM, MOMOTAZ *
ASSISTANT PROFESSOR
DEPARTMENT OF COMPUTER SCIENCE
UNIVERSITY OF MASSACHUSETTS LOWELL
LOWELL, MA 01234

BUDDE, MATTHEW D *
MILWAUKEE VA MEDICAL CENTER
ASSISTANT PROFESSOR OF NEUROSURGERY
MEDICAL COLLEGE OF WISCONSIN
MILWAUKEE, WI 53295

CULLUM, MUNRO, PHD *
PAMELA BLUMENTHAL DISTINGUISHED PROFESSOR OF PSYCHIATRY
PAMELA BLUMENTHAL DISTINGUISHED PROF CLIN PSYCH
CHIEF OF PSYCHOLOGY
DIRECTOR OF NEUROPSYCHOLOGY
UNIV OF TEXAS SW MEDICAL CENTER
DALLAS, TX 75390

DREBING, CHARLES E, PHD *
MENTAL HEALTH SERVICE LINE MANAGER
EDITH NOURSE ROGERS MEM VET HOSP
ASSOCIATE DIRECTOR OF VISN 1 NEW ENGLAND
BEDFORD VA MEDICAL CENTER
BEDFORD, MA 01730

DRONKERS, NINA F., PHD *
DIRECTOR/ADJUNCT PROFESSOR
CENTER FOR APHASIA & RELATED DISORDERS
VA NORTHERN CALIFORNIA HEALTH CARE SYSTEM
MARTINEZ, CA 94553

FISCHER, ELLEN P, PHD *
LITTLE ROCK VA MEDICAL CENTER
ASSOCIATE PROFESSOR
DIVISION OF MENTAL HLTH SERVICE RES.
DEPARTMENT OF PSYCHIATRY
UNIVERSITY ARKANSAS FOR MED SCIENCES
LITTLE ROCK, AR 72204

FREGNI, FELIPE, PHD, MD *
ASSOCIATE PROFESSOR OF PHYSICAL MEDICINE AND REHABILITATION
ASSOCIATE PROFESSOR OF NEUROLOGY
HARVARD MEDICAL SCHOOL
BOSTON, MA 02214

GARD, STEVEN A., PHD *
DIRECTOR
JESSE BROWN VA MEDICAL CENTER
ASSOC PROF IN PHYSICAL MEDICINE AND REHAB
NORTHWESTERN UNIVERSITY
CHICAGO, IL 60611

GOODMAN, MARIANNE, MD *
JAMES J. PETERS VA MEDICAL CENTER
PSYCHIATRIST
DEPARTMENT OF PSYCHIATRY
MOUNT SINAI SCHOOL OF MEDICINE
BRONX, NY 10468

GRABOWSKI, ALENA, PHD *
RESEARCH ASSISTANT
RESEARCH SERVICE
VA EASTERN COLORADO HEALTH CARE SYSTEM
DENVER, CO 80220

HANSEN, ANDREW H., PHD *
RESEARCH BIOMEDICAL ENGINEER
EXTENDED CARE AND REHABILITATION
MINNEAPOLIS VA HEALTH CARE SYSTEM
PROGRAM IN REHABILATION SCIENCE
UNIVERSITY OF MINNESOTA
MINNEAPOLIS, MN 55417

HEAPY, ALICIA, PHD *
ASSOCIATE DIRECTOR
RESEARCH ENHANCEMENT AWARD PROGRAM
VA CONNECTICUT HEALTH CARE SYSTEM - WEST HAVEN
ASSISTANT PROFESSOR PSYCHOLOGY
YALE SCHOOL OF MEDICINE
WEST HAVEN, CT 06516

HULA, WILLIAM D., PHD *
SPEECH PATHOLOGIST
GERIATRIC RESEARCH, EDUCATION & CLINICAL CENTER
VA PITTSBURGH HEALTHCARE SYSTEM
PITTSBURGH, PA 15240

KEAN, JACOB T., PHD *
HEALTH RESEARCH SCIENTIST
HSR&D CIEBP
RICHARD L. ROUDEBUSH VA MEDICAL CENTER
ASSIST PROF, DEPT OF PHYSICAL MED & REHAB
INDIANA UNIVERSITY SCHOOL OF MEDICINE
INDIANAPOLIS, IN 46202

KERN, ROBERT S, PHD *
RESEARCH PSYCHOLOGIST
VA GREATER LA HEALTHCARE CENTER
UCLA DEPT OF PSYCHIATRY AND BIOBEHAVIORAL
SCIENCES
DAVID GEFFEN SCHOOL OF MEDICINE
LOS ANGELES, CA 90073

KING, LAURIE, PHD *
VA PORTLAND HEALTH CARE SYSTEM
ASSISTANT PROFESSOR OF NEUROLOGY
OREGON HEALTH & SCIENCES UNIVERSITY
PORTLAND, OR 97239

LEE, THAY Q., PHD *
SENIOR RESEARCH CAREER SCIENTIST
VA LONG BEACH HEALTHCARE SYSTEM
UCI MEDICAL CENTER, ORTHOPAEDIC SURGERY
UNIVERSITY OF CALIFORNIA
LONG BEACH, CA 90822

LEPAGE, JAMES, PHD *
ACOS FOR RESEARCH AND DEVELOPMENT
VA NORTH TEXAS HEALTHCARE SYSTEM, DALLAS VAMC
DALLAS, TX 75216

LEVY, CHARLES EDWARD, MD *
CHIEF OF PM&R
VA CTR OF INNOVATION FOR DISABILITY AND REHAB RES
NORTH FLORIDA/SOUTH GEORGIA VETERANS HLTH SYST
ASSOCIATE PROFESSOR, REHABILITATION SCIENCE
UNIVERSITY OF FLORIDA
GAINESVILLE, FL 32608

MAHER, LYNN M., PHD *
HOUSTON VA MEDICAL CENTER
PROFESSOR AND CHAIR
DEPARTMENT OF COMMUNICATION SCIENCES AND
DISORDERS
UNIVERSITY OF HOUSTON
HOUSTON, TX 77030

MCCULLY, KEVIN K., PHD *
PROFESSOR
DEPARTMENT OF KINESIOLOGY
UNIVERSITY OF GEORGIA
ATHENS, GA 30602

MOREY, MIRIAM C., PHD *
ASSOCIATE DIRECTOR OF RESEARCH
GRECC, DURHAM VA MEDICAL CENTER
PROFESSOR IN MEDICINE
DEPT OF MEDICINE, DIVISION OF GERIATRICS
DUKE UNIVERSITY SCHOOL OF MEDICINE
DURHAM, NC 27705

MURPHY, SUSAN L., SCD *
ASSOCIATE PROFESSOR AND DIRECTOR OF CLINICAL
TRIALS DEVELOPMENT
PHYSICAL MEDICINE AND REHABILITATION DEPARTMENT
REARCH HEALTH SCIENCE SPECIALIST
VA ANN ARBOR HEALTH CARE SYSTEM GRECC
UNIVERSITY OF MICHIGAN
ANN ARBOR, MI 48105

MYERS, JONATHAN N., PHD *
HEALTH RESEARCH SCIENTIST
VA PALO ALTO HEALTH CARE SYSTEM
PALO ALTO, CA 94304

NOCERA, JOE ROBERT, PHD *
HEALTH SCIENCE SPECIALIST
ATLANTA VA MEDICAL CENTER
ASSISTANT PROFESSOR
DEPARTMENT OF NEUROLOGY
EMORY UNIVERSITY SCHOOL OF MEDICINE
ATLANTA, GA 30322

ROBINSON, THOMAS NICHOLS, MD *
CHIEF OF SURGERY
DENVER VA MEDICAL CENTER
PROFESSOR
UNIVERSITY OF COLORADO
AURORA, CO 80045

ROSEN, CRAIG S *
NATIONAL CENTER FOR PTSD
VA PALO ALTO HEALTH CARE SYSTEM (334-PTSD)
ASSOC PROF OF PSYCHIATRY AND BEHAVIORAL SCIENCES
STANFORD UNIVERSITY
MENLO PARK, CA 94025

SOMERS, TAMARA J, PHD *
CLINICAL PSYCHOLOGIST AND ASSISTANT PROFESSOR OF
PSYCIATRY AND BEHAVIORL SCIENCES
DUKE UNIVERSITY
DURHAM, NC 27705

STROHL, KINGMAN PERKINS, MD *
PROFESSOR AND PROGRAM DIRECTOR
CLEVELAND VA MEDICAL CENTER
DIVISION OF PULMONARY, CRITICAL CARE
AND SLEEP MEDICINE
UNIVERSITY HOSPITALS OF CLEVELAND
CLEVELAND, OH 44106

SCIENTIFIC REVIEW OFFICER

SCHULZ, BRIAN W., PHD
RESEARCH BIOMEDICAL ENGINEER
DEPARTMENT OF VETERANS AFFAIRS
OFFICE OF RESEARCH AND DEVELOPMENT
REHABILITATION RESEARCH AND DEVELOPMENT SERVICE
WASHINGTON, DC 20240

* Temporary Member. For grant applications, temporary members may participate in the entire meeting or may review only selected applications as needed.

Consultants are required to absent themselves from the room during the review of any application if their presence would constitute or appear to constitute a conflict of interest.
